# Case Report: Multimodal ultrasound diagnosis of traumatic right coronary sinus rupture combined with atrial septal tears

**DOI:** 10.3389/fmed.2026.1812111

**Published:** 2026-07-03

**Authors:** Jinting Wang, Cui Yang, Yeshun Wu

**Affiliations:** 1 Department of Ultrasound, Quzhou People’s Hospital, The Quzhou Affiliated Hospital, Wenzhou Medical University, Quzhou, China; 2 Department of Cardiology, Quzhou People’s Hospital, The Quzhou Affiliated Hospital, Wenzhou Medical University, Quzhou, China

**Keywords:** atrial septal tears, cardiac injury, case report, multimodal ultrasound, polytrauma, right coronary sinus rupture

## Abstract

**Background:**

Cardiac injury in patients with severe polytrauma is often occult and carries a high mortality, making rapid and accurate diagnosis a significant clinical challenge.

**Case presentation:**

A 57-year-old Asian male was admitted with polytrauma and impaired consciousness for over 2 h after a fall from the 9th floor. Post-injury manifestations included confusion, restlessness, and active bleeding from multiple wounds. The initial diagnosis was polytrauma with traumatic hemorrhagic shock. Following initial resuscitation and thoracic endovascular aortic repair (TEVAR), he developed refractory hypoxemia and hemodynamic instability. Subsequent multimodal ultrasound revealed combined injuries: traumatic rupture of the right coronary sinus into the right atrium and right ventricle, multiple tears in the mid-portion of the atrial septum, and bidirectional shunt at the atrial level. The patient received comprehensive treatment, including TEVAR, mechanical ventilation, anti-infection therapy, maintenance of internal homeostasis, and nutritional support. Due to cardiopulmonary failure secondary to the cardiac injury, extracorporeal membrane oxygenation (ECMO) was initiated. Despite aggressive life-sustaining measures, the patient developed severe infection and multiple organ failure, precluding cardiac surgical intervention. He ultimately succumbed after the withdrawal of ECMO support.

**Conclusion:**

In the management of polytrauma, a high index of suspicion for occult cardiac injuries is essential. Multimodal echocardiography—comprising transthoracic echocardiography, transesophageal echocardiography, and agitated saline contrast echocardiography—serves as an efficient and precise bedside diagnostic toolkit. It not only enables rapid diagnosis of complex intracardiac injuries and hemodynamic assessment but also plays a crucial role in guiding advanced life support and developing effective treatment strategies. The early and systematic application of this approach may facilitate timely intervention for critically ill patients with polytrauma.

## 1 Introduction

Cardiac injury, though uncommon in polytrauma, is often clinically occult and associated with high mortality. Its rapid and accurate diagnosis remains a significant clinical challenge (1–3). In recent years, multimodal ultrasonography—integrating transthoracic echocardiography (TTE), transesophageal echocardiography (TEE), and agitated saline contrast echocardiography (ASCE)—has emerged as a promising core tool for diagnosing cardiac injuries and developing treatment strategies, owing to its bedside availability, speed, non-invasiveness, and repeatability (4–7). It not only allows for prompt assessment of cardiac function but also enables comprehensive screening for structural and hemodynamic abnormalities. This report presents a rare and occult case of right coronary sinus rupture combined with atrial septal tears, aiming to highlight the diagnostic value of multimodal ultrasonography in such complex critical injuries and to provide a reference for clinical practice in the future.

## 2 Case description

A 57-year-old Asian male was admitted with multiple traumas and impaired consciousness for over 2 h after a high-altitude fall. According to his wife, he had no reported history of hypertension, diabetes, or cardiovascular/cerebrovascular diseases. No family history of connective tissue disorders or congenital heart disease was reported. He consumed alcohol occasionally and had smoked approximately 10 cigarettes daily for over 20 years.

**Figure 1:**
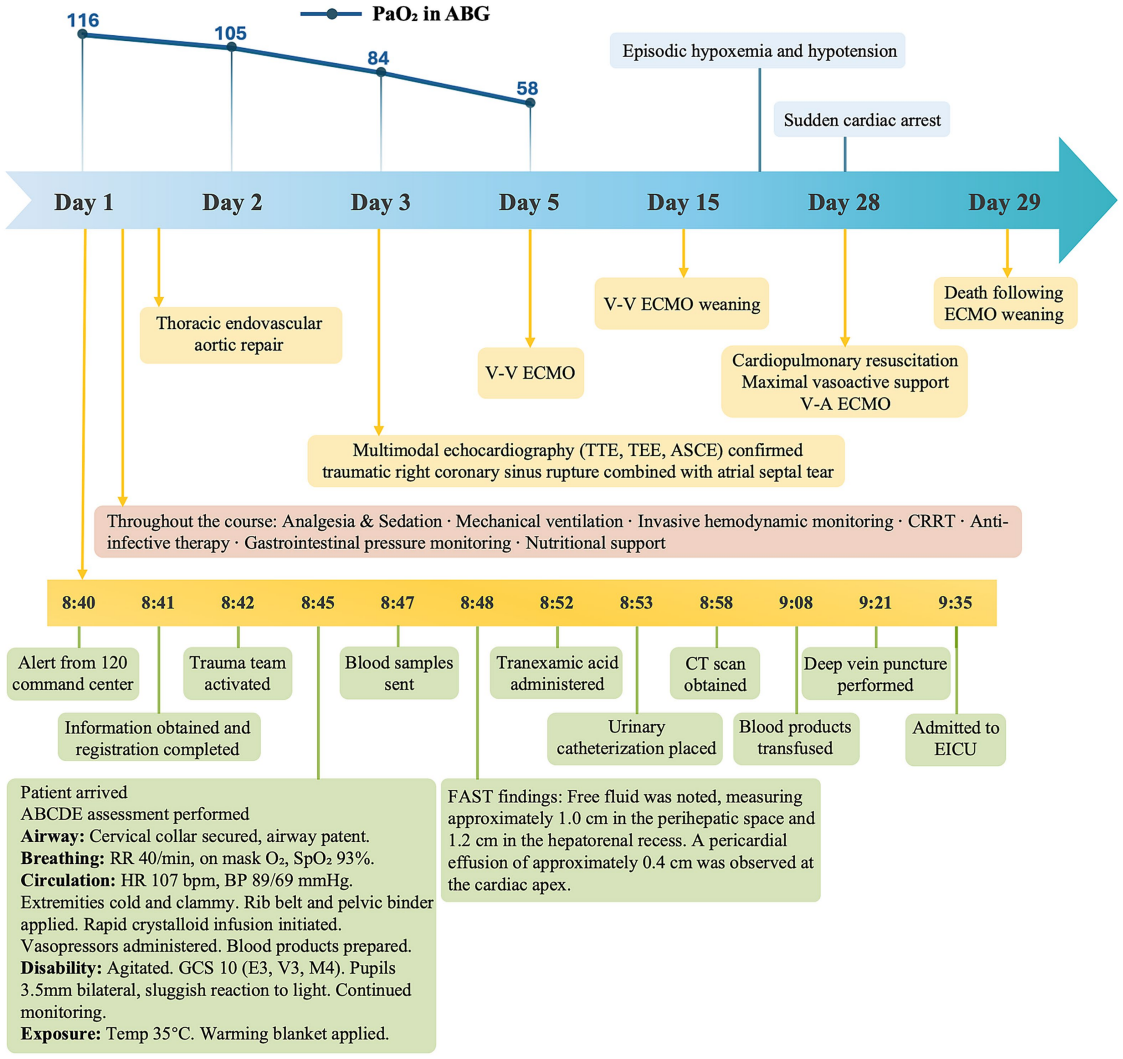
Case progress timeline. ABG, arterial blood gas; ASCE, agitated saline contrast echocardiography; BP, blood pressure; CRRT, continuous renal replacement therapy; CT, computed tomography; ECMO, extracorporeal membrane oxygenation; EICU, emergency intensive care unit; FAST, focused assessment with sonography for trauma; GCS, Glasgow coma scale; HR, heart rate; RR, respiratory rate; TEE, transesophageal echocardiography; TEVAR, thoracic endovascular aortic repair; TTE, transthoracic echocardiography.

**Figure 2:**
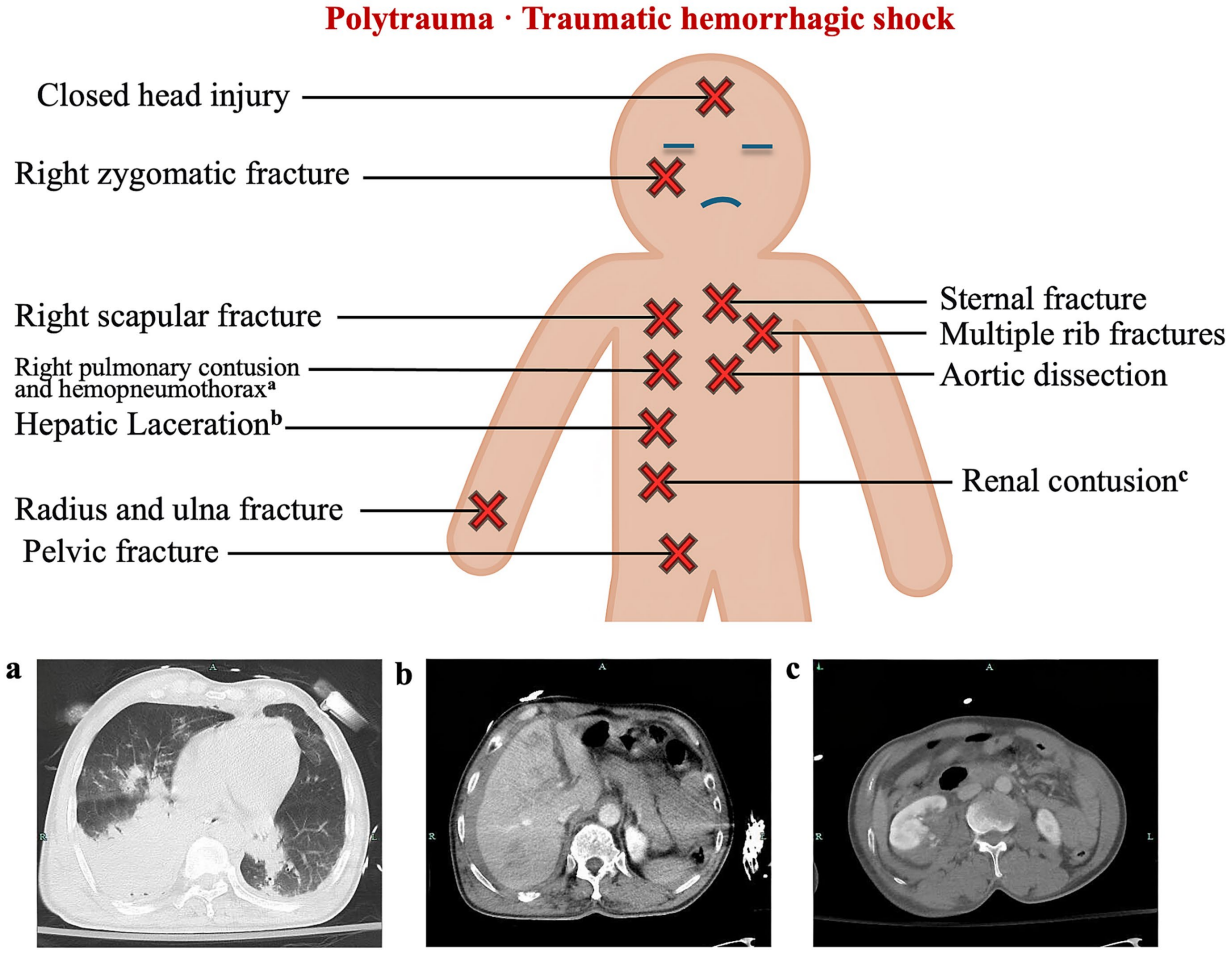
Schematic diagram of trauma location.

**Figure 3:**
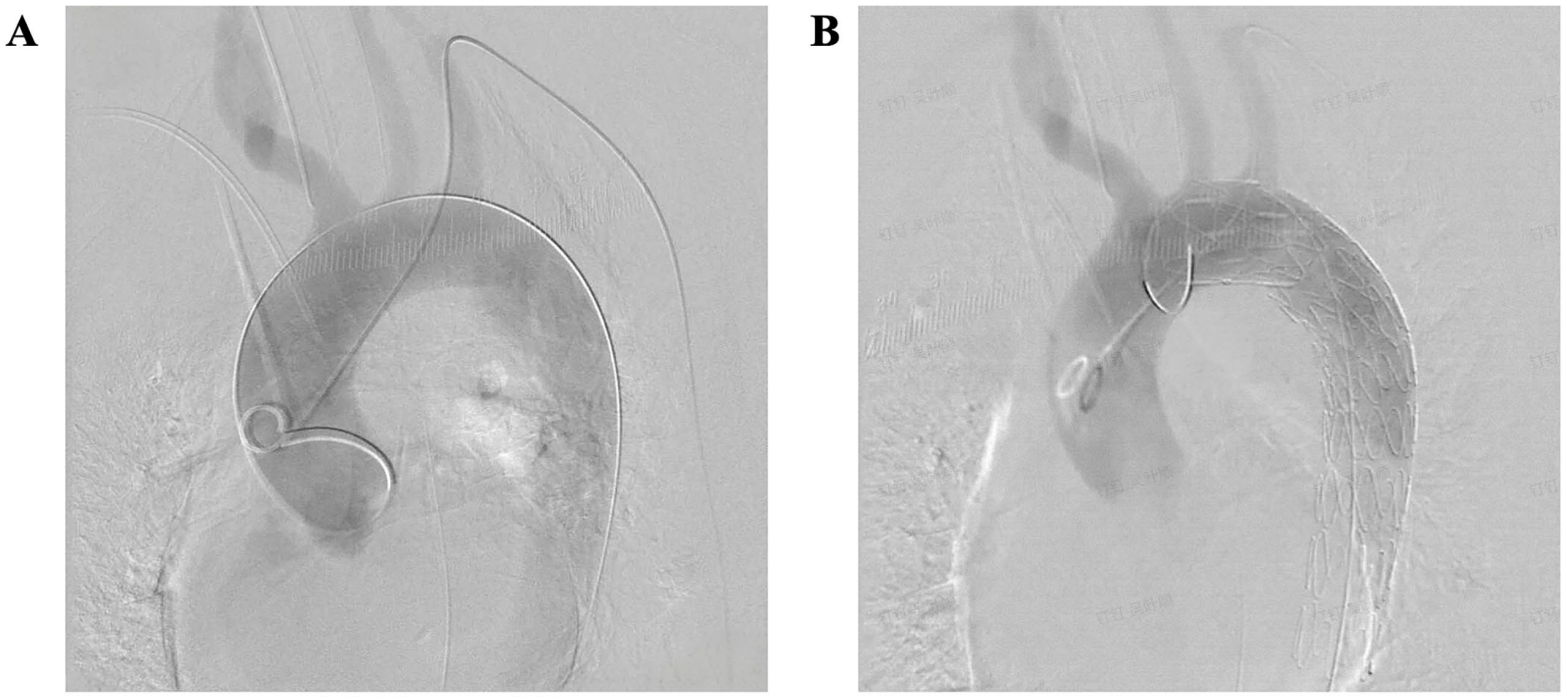
Aortic dissection and thoracic endovascular aortic repair. **(A)** Angiography showing aortic dissection. **(B)** Postoperative image after thoracic endovascular aortic repair.

Two hours prior to admission, the patient fell from the 9th floor onto muddy soil, sustaining multiple injuries with oozing wounds. He presented with confusion and restlessness. Upon early warning notification from the emergency command center, the trauma team was immediately activated, performing an ABCDE assessment and focused assessment with sonography for trauma (FAST) (Figure 1). The initial diagnosis was polytrauma and traumatic hemorrhagic shock (Figure 2). After multidisciplinary discussion, thoracic endovascular aortic repair (TEVAR) was performed (Figure 3). Postoperative management continued with mechanical ventilation, anti-infection therapy, maintenance of homeostasis, and nutritional support.

**Figure 4:**
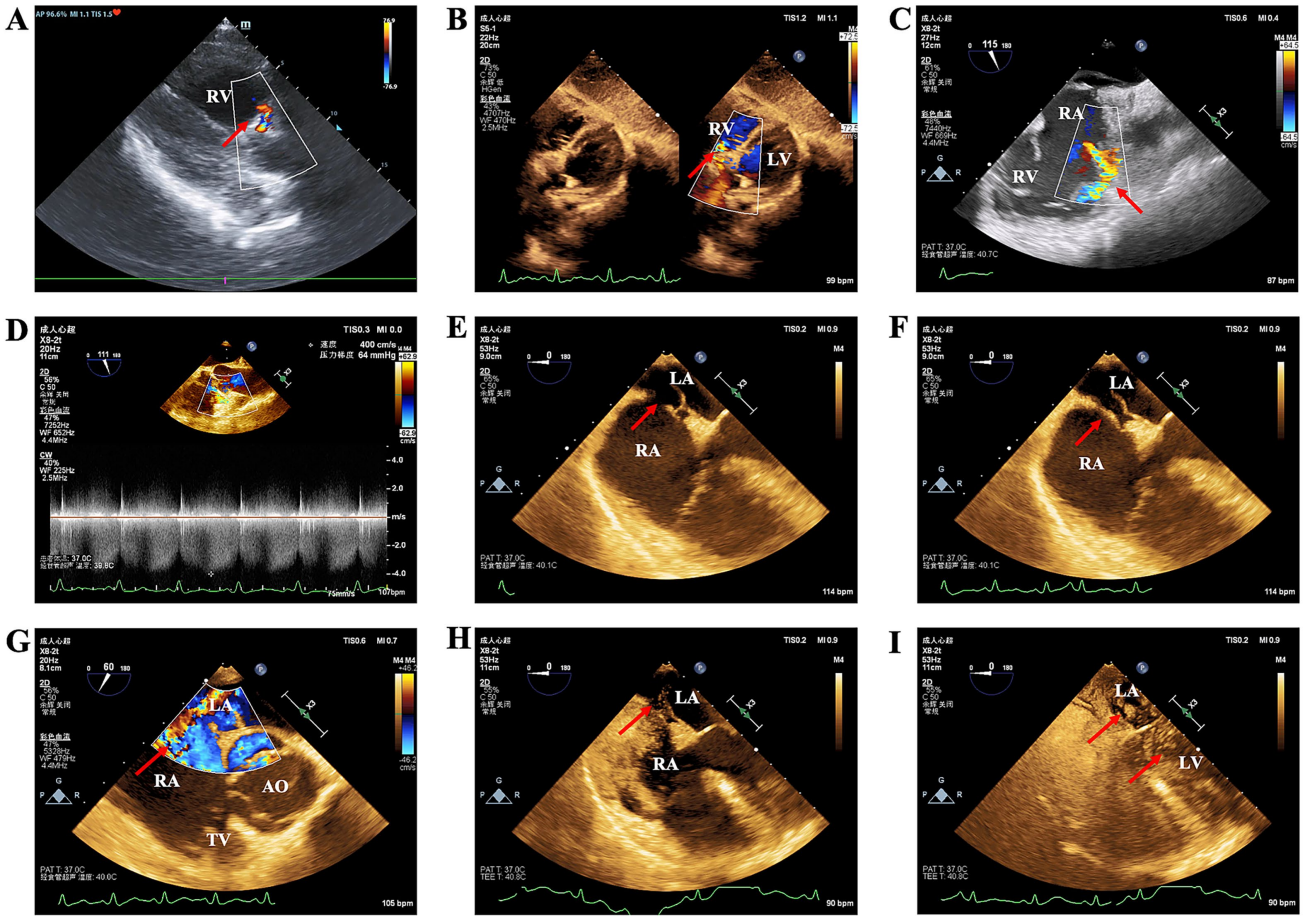
Multimodal echocardiography confirmed traumatic right coronary sinus rupture combined with atrial septal tear. **(A)** TTE revealed an abnormal flow jet from the right coronary sinus shunting into the right ventricle. **(B)** TTE revealed a similar abnormal shunt in the subxiphoid view. **(C)** TEE showed the rupture of the right coronary sinus into both the right atrium and right ventricle. **(D)** TEE exhibited a pansystolic and pandiastolic shunt on color Doppler with a velocity of 400 cm/s and a maximum pressure gradient of 64 mmHg. **(E,F)** TEE showed the separation of the two atrial septal layers, with a flail flap bulging and fluttering into the left atrium. **(G)** TEE exhibited a bidirectional shunt at the atrial level on color Doppler. **(H,I)** ASCE demonstrated rapid left-heart opacification after contrast agent entered the right heart, with numerous microbubbles crossing from the atrial level into the left atrium and subsequently the left ventricle. ASCE, agitated saline contrast echocardiography; TEE, transesophageal echocardiography; TTE, transthoracic echocardiography.

Subsequently, the patient’s oxygenation continued to decline (Figure 1). Repeat bedside TTE on day 3 revealed an abnormal flow jet from the right coronary sinus shunting into the right ventricle (Figure 4A), a similar abnormal shunt in the subxiphoid view (Figure 4B), right ventricular enlargement, degenerative aortic valve disease with increased transvalvular flow velocity, trivial-to-mild tricuspid regurgitation, and mild pulmonary hypertension. These findings suggested the rupture of the right coronary sinus into the right ventricle. For definitive diagnosis, TEE was performed, showing: rupture of the right coronary sinus into both the right atrium and right ventricle (Figure 4C), which on color Doppler exhibited a pansystolic and pandiastolic shunt with a velocity of 400 cm/s and a maximum pressure gradient of 64 mmHg (Figure 4D); separation of the two atrial septal layers, with a flail flap bulging and fluttering into the left atrium (Figures 4E,F); and a bidirectional shunt at the atrial level on color Doppler (Figure 4G). ASCE further demonstrated rapid left-heart opacification after contrast agent entered the right heart, with numerous microbubbles crossing from the atrial level into the left atrium and subsequently the left ventricle (Figures 4H,I). The final diagnosis was traumatic rupture of the right coronary sinus into the right atrium and right ventricle, multiple tears in the mid-portion of the atrial septum, and a bidirectional atrial-level shunt.

Due to severe concomitant infection and multiple organ failure, cardiac surgery was not immediately feasible. Veno-venous extracorporeal membrane oxygenation (ECMO) support was initiated on day 5 and discontinued on day 15. However, the patient continued to experience episodes of hypoxemia and hypotension. On day 28, he suffered sudden cardiac arrest. Following resuscitation, heart rate and blood pressure were restored. After evaluation, maximal vasoactive support and veno-arterial ECMO were initiated. Nevertheless, severe infection and multiple organ failure precluded any window for surgical repair of the cardiac rupture. After multidisciplinary discussion and in accordance with the family’s wishes, ECMO was withdrawn on day 29, and the patient died (Figure 1).

## 3 Discussion

This case involved polytrauma from a high-altitude fall, presenting an extremely complex and critical condition. The diagnosis was ultimately established through the synergistic application of multimodal echocardiography (TTE, TEE, and ASCE), which identified the rare and fatal combined cardiac injury: traumatic rupture of the right coronary sinus into the right atrium and right ventricle, accompanied by multiple atrial septal tears. Although the patient ultimately succumbed due to an inability to tolerate surgical repair of the cardiac rupture, the diagnostic process underscores the indispensable value of the multimodal ultrasound, particularly in patients with refractory hypoxemia and hemodynamic instability.

In patients with polytrauma, routine TTE reveals concomitant right ventricular dysfunction in a significant proportion, with wall motion abnormalities in 25% and relaxation dysfunction in 20% (8). Severe blunt chest and abdominal injuries are frequently associated with cardiac and major vascular damage. Previous studies indicate that the incidence of blunt cardiac rupture (BCR) among trauma-related admissions is approximately 0.16 to 2%, while traumatic aortic dissection (TAD) accounts for about 1.79% of blunt chest trauma cases. Although not predominant in incidence, their mortality is exceedingly high: 91% of patients with BCR die within 30 min of injury, and 85% of patients with TAD die before reaching the hospital (9, 10). When the chest sustains a sudden severe impact, intrathoracic pressure rises sharply, creating high intracardiac and intravascular pressures that can lead to rupture at vulnerable sites and valvular injury (10). In cases of falls from height, the “water hammer” effect makes the right atrium one of the most commonly injured sites (11). In the acute phase, such injuries are often masked by or attributed to other more obvious trauma (e.g., hemopneumothorax or intra-abdominal bleeding) due to impaired consciousness and hemodynamic instability, leading to missed diagnosis (1, 10). Aortic sinus rupture and atrial septal tear, as relatively rare forms of BCR, may be partially compensated initially by systemic hypovolemia and low cardiac output, resulting in atypical clinical signs that pose significant challenges for early identification (1, 12). In this case, although computed tomography suggested abnormalities near the aortic arch, and bedside FAST with TTE revealed pericardial effusion, no direct evidence of specific intracardiac rupture was initially captured, illustrating the limitations of a single modality in diagnosing complex cardiac injuries.

The successful diagnosis in this case illustrates an effective multimodal, stepwise ultrasound strategy: using TTE for initial screening, TEE as the core tool for precise anatomical definition, and ASCE for functional verification. TTE, being bedside, non-invasive, and repeatable, is the first-choice screening tool for suspected cardiac injury. However, patients with trauma are often have positioning limitations or associated conditions like pneumothorax that can impair imaging quality (13, 14). In this patient, initial FAST with TTE did not detect the aortic sinus rupture or atrial septal tear, but it did identify pericardial effusion—a crucial warning sign suggesting potential cardiac or aortic root injury, thereby directing closer monitoring of cardiac structures. When TTE findings are suspicious, TEE becomes irreplaceable for accurate diagnosis. Positioned close to the posterior heart, the TEE probe avoids interference from the chest wall and lung, providing high-resolution images superior to TTE (15, 16). A meta-analysis by Kyriazidis et al. reported a sensitivity of 93.1% (95% CI: 76.5–98.2) for TEE in diagnosing cardiac injury, compared to 55.1, 64.4, 68.4, 55.2, and 47.0% for electrocardiograph, Troponin I, Troponin T, creatine kinase-MB, and TTE, respectively (17). Furthermore, after TEE identified the right coronary sinus rupture and atrial septal tears, ASCE was performed for functional verification and shunt localization. As a functional assessment, ASCE complements the anatomical information from TEE. Together, from both morphological and functional perspectives, they confirmed the diagnosis and visualized the pathophysiological consequences of the shunt, such as hemodynamic disturbances and hypoxemia (18–20).

Beyond its diagnostic utility, multimodal echocardiography played a pivotal role in guiding the complex therapeutic trade-offs in this polytrauma patient with occult cardiac injuries. Initially, refractory hypoxemia constituted the dominant life-threatening challenge. Veno-venous ECMO was selected to provide respiratory support while avoiding increased left ventricular afterload. This configuration is particularly advantageous when a left-to-right shunt is present as in this case, because veno-arterial ECMO could theoretically exacerbate the diversion through increased pulmonary congestion. When the patient subsequently suffered cardiac arrest, the hemodynamic profile had evolved to overt cardiogenic shock, necessitating conversion to veno-arterial ECMO. Regarding definitive repair, prompt surgical correction under cardiopulmonary bypass remains the ideal treatment for both right coronary sinus rupture and traumatic atrial septal tears. However, at the time of diagnosis, this patient presented multiple contraindications to surgery, including severe infection, multiple organ failure, and hemodynamic fragility. Multidisciplinary consensus concluded that cardiopulmonary bypass was not tolerable. Although percutaneous closure of the atrial septal tear was contemplated as a less invasive alternative, this approach was deemed technically infeasible because the tears were multiple, the septal tissue was flail, and the coronary sinus rupture could not be addressed percutaneously. Consequently, ECMO was employed as a bridge to potential recovery or further decision -making. Nevertheless, the patient’s overall trajectory continued downward due to uncontrolled infection and progressive organ failure. The therapeutic objectives thus evolved through distinct phases: from initial damage-control resuscitation and life-saving TEVAR, to cardiopulmonary stabilization with ECMO aimed at heart and lung recovery, and ultimately to recognition of futility upon development of irreversible multiple organ failure, leading to withdrawal of ECMO in accordance with the family’s wishes after thorough communication. This case underscores that in severe polytrauma, even with a precise anatomical diagnosis, the window for surgical repair may be precluded by systemic deterioration. Nonetheless, early multimodal echocardiography remains indispensable for guiding clinical decision-making at each critical juncture.

TTE, TEE, and ASCE are not applied in isolation but form an integrated diagnostic chain. TTE provides initial clues, TEE precisely locates anatomical abnormalities, and ASCE verifies functional significance. This integrated multimodal approach offers unique advantages in severe polytrauma: (1) High Timeliness: All can be performed immediately at the bedside or in the operating room; (2) High Accuracy: Excellent spatial and temporal resolution for intracardiac structures and flow abnormalities, potentially serving as the gold standard for diagnosing intracardiac shunts, valvular regurgitation, and cardiac function (21–23); (3) Dynamic Assessment: Allows real-time, continuous monitoring of disease progression, including pericardial effusion volume, shunt status, cardiac function, and so on, facilitating guidance for fluid resuscitation and advanced life support (24–27); and (4) Safety: Non-radioactive, safe contrast agents, and no need to move critically ill patients, aligning with damage-control principles.

The regret in this case is the patient’s excessively severe systemic trauma and depleted physiological reserve, which never allowed him to reach tolerable for crucial surgical repair of the cardiac rupture. This underscores the need for earlier multidisciplinary intervention in such critically ill patients. For suspected cases, utilizing multimodal ultrasound for rapid and clear diagnosis as early as possible, and considering timely damage-control procedures (e.g., atrial septal closure) under ECMO support may be worthwhile in future cases.

## 4 Conclusion

Traumatic right coronary sinus rupture combined with atrial septal tears is a rare and life-threatening cardiac injury. This case demonstrates that in the management of severe polytrauma, clinicians should maintain a high level of suspicion for occult cardiac injuries. The sequential and integrated application of multimodal echocardiography (TTE, TEE, and ASCE) constitutes an effective toolkit for rapid, accurate, and bedside diagnosis of such complex intracardiac injuries. It not only clarifies the anatomical abnormalities but also reveals their hemodynamic consequences, which holds irreplaceable core value in explaining clinical symptoms, guiding advanced life support (e.g., ECMO), and developing effective treatment strategies.

## Data Availability

The original contributions presented in the study are included in the article/supplementary material, further inquiries can be directed to the corresponding author.
